# An integrated approach for identification of a panel of candidate genes arbitrated for invasion and metastasis in oral squamous cell carcinoma

**DOI:** 10.1038/s41598-021-85729-x

**Published:** 2021-03-18

**Authors:** Samapika Routray, Ravindra Kumar, Keshava K. Datta, Vinuth N. Puttamallesh, Aditi Chatterjee, Harsha Gowda, Neeta Mohanty, Rupesh Dash, Anshuman Dixit

**Affiliations:** 1 Department of Dentistry, All India Institute of Medical Sciences, Bhubaneswar, 751019 India; 2 School of Biotechnology, National Institute of Technology Calicut, Kozhikode, 673601 Kerala India; 3 QIMR Berghofer Medical Research Institute, Brisbane, Australia; 4 Institute of Bioinformatics, Bangalore, India; 5 Amrita School of Biotechnology, Amrita Vishwa Vidyapeetham, Kollam, India; 6 Manipal Academy of Higher Education, Madhav Nagar, Manipal, India; 7 Department of Oral Pathology & Microbiology, Institute of Dental Sciences, Siksha ‘O’ Anusandhan (Deemed to be) University, Bhubaneswar, India; 8 Institute of Life Sciences, Bhubaneswar, India

**Keywords:** Oral cancer, Transcriptomics, Bioinformatics, Mass spectrometry, Immunohistochemistry

## Abstract

Oral squamous cell carcinoma (OSCC) is known for its aggressiveness associated with poor prognosis. The molecular mechanisms underlying the invasion and metastasis are still poorly understood. An improved understanding of these mechanisms shall precede the development of new diagnostic tools and targeted therapies. We report an integrated approach using bioinformatics to predict candidate genes, coupled with proteomics and immunohistochemistry for validating their presence and involvement in OSCC pathways heralding invasion and metastasis. Four genes POSTN, TNC, CAV1 and FSCN1 were identified. A protein–protein interaction network analysis teamed with pathway analysis led us to propose the role of the identified genes in invasion and metastasis in OSCC. Further analyses of archived FFPE blocks of various grades of oral cancer was carried out using TMT-based mass spectrometry and immunohistochemistry. Results of this study expressed a strong communiqué and interrelationship between these candidate genes. This study emphasizes the significance of a molecular biomarker panel as a diagnostic tool and its correlation with the invasion and metastatic pathway of OSCC. An insight into the probable association of CAF's and these biomarkers in the evolution and malignant transformation of OSCC further magnifies the molecular-biological spectrum of OSCC tumour microenvironment.

## Introduction

Oral squamous cell carcinoma (OSCC), amidst all head and neck squamous cell carcinoma (HNSCC), is the most commonly occurring cancer, predominantly seen in males. As per the data stated by World Health Organization (WHO’s) International Agency for Research in Cancer (IARC) (https://gco.iarc.fr/tomorrow/). The estimated number of cases of lip and oral cavity cancer in both sexes shall increase to 5,53,000 by 2040 worldwide. In males only the estimated number incident cases shall be 3,82,000 by 2040^1^. OSCC often goes unnoticed until advanced stages and is associated with a high mortality rate, bringing substantial personal and societal costs. The current understanding of the molecular basis of aggressive OSCC invasion and metastasis is still imprecise. Focusing on epithelial tumours, break in the continuity of the basement membrane (BM) is critical for invasion into the stroma. Although metastasis occurs by motility of tumour cells^2^; it comprises of a series of sequential and interrelated steps such as an invasion of extracellular matrix; angiogenesis, vascular dissemination and anoikis resistance; tumour homing and relocation of tumour cells to the selective organ are crucial^3^. Carcinoma associated fibroblasts (CAFs) in the tumour microenvironment (TME) are known to secrete a plethora of factors. In-turn this induces a more aggressive TME phenotype^4^. Considering that metastasis has been known to be a bad prognosticator in cancer and understanding the molecular basis of invasion and metastasis may help predict aggressive behaviour of OSCC, this study focussed on discovering a panel of invasion and metastasis related biomarkers^5,6^. Rivera et al. in 2017; in his analysis found forty-one biomarkers, mostly proteins evaluated by immunohistochemistry (IHC)^7^. Looking for prognostic biomarkers for oral tongue squamous cell carcinoma (OTSCC) to predict better tumour behaviour too yielded five biomarkers following the REMARK guidelines^8^. In recent times studies with "omics"-based integrated approaches have helped as tools to screen relevant changes in oral cancer and assess biomarkers at a molecular level^9^. Based on similar approaches^10^, we proposed this study for identifying and assessing invasive and metastatic candidate biomarkers of OSCC.

## Material and methods

In this study, we aimed to unveil a panel of candidate genes involved in OSCC pathogenesis using a multi-integrated approach. The study protocol was approved by Institutional Ethics Committee, IMS & SUM Hospital, Siksha ‘O’ Anusandhan University, Bhubaneswar and was in accordance to the Declaration of Helsinki and written informed consent was obtained from all the participant as per the requisite.

### Bioinformatic analysis

In our study, to know the metastatic genes, HCMDB (Human Cancer Metastasis Database), an integrated database was searched (https://hcmdb.i-sanger.com/). Oral cancer-related genes were collected from three other databases viz. Head-Neck and Oral Cancer Database (HNOCDB) v1 (http://gyanxet.com/hno.html), Oral Cancer Gene Database (OrCGDB) v2 (http://www.actrec. gov.in/OCDB/index.htm) and the Head and Neck database (HNdb) (http://www.gencapo.famerp.br/hndb/). The jVenn software was used to identify collective genes of metastatic origin amongst these databases^11^. We further investigated the predicted genes using evidence from literature, protein–protein interaction (PPI) network analysis through Search Tool for the Retrieval of Interacting Genes/Proteins (STRING), databases like Oncomine and Cancer RNA-Seq Nexus (CRN, http://syslab4.nchu.edu.tw/CRN) and identified four candidate genes viz. periostin (POSTN), tenascin (TNC), caveolin 1 (CAV1) and fascin1 (FSCN1).

### Proteomic analysis

Protein from five samples each of well, moderate, and poorly differentiated grades of OSCC cases were pooled for proteomic analysis. We employed pressure cycling technology (PCT) that has been demonstrated to be efficient for proteomic analysis of archived formalin fixed paraffin embedded (FFPE) biopsy samples^12^. The clinicopathological features of the samples used for proteomic study are provided in Table 1.

**Table 1:** Clinicopathological features of samples of OSCC for POSTN, TNC, CAV1, FSCN1 for Proteomic analysis.

Variables	Numbers (n)
**Age**	
≤ 50 years	06
> 50 years	09
**Sex**	
Male	09
Female	06
**Habit**	
Chewing	11
Chewing + smoking	03
Chewing + smoking + alcohol	01
**Site**	
Gingivo-buccal complex (GBC)	12
Buccal/labial mucosa	03
**Tumor staging (TNM) [according 7th ed. TNM]**	
I	01
II	03
II	03
IV	08
**Differentiation (grading)**	
Well	05
Moderate	05
Poor	05

#### Protein extraction and digestion using pressure cycling technology

Protein extraction from FFPE sections and protein digestion was performed using Barocycler NEP2320 (Pressure BioSciences, Inc, South Easton, MA). Deparaffinised FFPE tissue sections were gently scraped off the glass slide and transferred to PCT microtubes. Around 150 µl of tissue lysis buffer (4% SDS, 100 mM DTT and 50 mM TEABC) was added and incubated at 95º for 10 min. Protein extraction was done at 95 ºC and 60 cycles of alternating pressure consisting of 50 s at 40,000 psi and 10 s at 5000 psi). Protein lysate was clarified at 12,000 rpm for 20 min and supernatant was transferred to separate tube. Equal amount of protein from each sample of well, moderate and poorly differentiated OSCC was pooled before protein digestion. Protein was reduced using 10 mM dithiothreitol (DTT) at 60 ºC for 30 min followed by alkylation using 20 mM iodoacetamide in dark for 10 min. PCT-based protein digestion was done using Lys-c and Trypsin. Briefly, Lys-C was added to the protein lysate at 1:100 enzyme to substrate ratio and transferred to PCT microtubes. Protein digestion was carried out at 32 ºC for 45 cycles with alternating pressure of 20,000 psi for 50 s and 5000 psi for 10 s. Following Lys-C digestion, trypsin was added at 1:50 enzyme to substrate ratio and digestion step was repeated using barocycler^13,14^.

#### TMT labelling and mass spectrometry data acquisition

TMT-labelling was carried out as per manufacturer’s protocol. Briefly, the lyophilized TMT-labels were reconstituted in 41 µl of anhydrous acetonitrile and added to the peptide samples. The reaction mixture was incubated for one hour and the reaction was quenched with 5% hydroxylamine. The samples were pooled, dried, desalted using STAGE tips, and dried.

The dried samples were reconstituted in 0.1% formic acid and analysed on an Orbitrap Fusion Tribrid mass spectrometer (ThermoFisher Scientific, Bremen, Germany). The samples were first loaded onto a trap column (75 µm × 2 cm, nanoViper, 3 µm, 100 Å) by an Easy-nLC-1200 at a flowrate of 4 µl/min and then resolved on an analytical column (15 cm × 50 µm, nanoViper, 2 μm). The mass spectrometer was operated in positive mode and data-dependent acquisition was carried out with Synchronous Precursor Selection (SPS-MS3) for TMT reporter ions. The maximum injection time was set to 200 ms while the automatic gain control value was 500,000. Higher energy collisional dissociation of the top ten most intense precursor ions was achieved by a normalized collision energy of 33%.

#### LC–MS/MS data analysis

The mass spectrometry data was searched against the Human Ref Seq 81 protein database using two search engines—SequestHT and Mascot, through the Proteome Discoverer software suite (version 2.1, Thermo Scientific, Bremen, Germany). Precursor mass tolerance was set to 10 ppm while the fragment mass tolerance was set to 0.05 Da. Search parameters consisted of trypsin as the proteolytic enzyme with a maximum of two allowed missed cleavages. Fixed modifications included carbamidomethylation of cysteine and TMT at N-termini of peptides and lysine. Dynamic modifications included acetylation of protein N-termini and oxidation of methionine. Protein quantitation was carried out using the reporter ion quantifier node. False Discovery Rate (FDR) was calculated using a Target-Decoy strategy and only those PSMs that cleared the FDR threshold of 1% were retained.

### Immunohistochemical analysis

Patient-related data extradited from archival records and haematoxylin and eosin (H&E) sections were reassessed for OSCC cases used from the archival FFPE blocks. Samples, 50 cases each (40 samples of OSCC and 10 Normal tissue) used for POSTN, TNC, CAV 1 with same clinical and demographic characteristics study group (group 1); whereas varying clinical and demographic characteristics were used for study group of FSCN1 (group 2).

The OSCC samples for group 1 consisted of 9, 15, 9 and 7 samples from Stage 1, Stage 2, Stage 3 and Stage 4 respectively. Grade wise, this group included 18, 12 and 10 samples of Grade 1 (Well differentiated), Grade 2 (Moderately differentiated) and Grade 3 (Poorly differentiated) respectively. The clinicopathological features of the samples used for this group are provided in Table 2.

**Table 2:** Clinical and demographic characteristics of the samples in OSCC for POSTN, TNC and CAV1 for Immunohistochemistry analysis.

Variables	Numbers (n)
**Age**	
(≥ 50)	27
(< 50)	23
**Sex**	
Male	35
Female	15
**Habit**	
No habit	10
Chewing	25
Alcohol	3
Smoking	1
Chewing + smoking	8
Chewing + alcohol	0
Smoking + alcohol	0
Chewing + alcohol + smoking	3
**Tumor location**	
Gingivobuccal complex	27
Buccal mucosa/labial mucosa	11
Tongue	03
Retro molar triangle	09
**Differentiation (grade)**	
No grade (normal mucosa)	10
Well	18
Moderate	12
Poor	10

The OSCC samples for group 2 consisted of 5, 9 and 26 samples from Stage 2, Stage 3 and Stage 4 respectively. Grade wise, this group included 18, 20 and 2 samples of Grade 1 (Well differentiated), Grade 2 (Moderately differentiated) and Grade 3 (Poorly differentiated) respectively. The clinicopathological features of the samples used for this group are provided in Table 3.

**Table 3:** Clinico-pathological features of samples of FSCN1 for Immunohistochemistry analysis.

Variables	Numbers (n)
**Age**	
≤ 50 years	29
> 50 years	21
**Sex**	
Male	27
Female	23
**Tobacco use (smokeless tobacco)**	
Yes	40
No	10
**Site**	
Gingivo-buccal complex (GBC)	40
**Tumor staging (TNM) [according 7th ed. TNM]**	
No stage	10
II	05
III	09
IV	26
**Local extension: tumor size (cT)**	
No tumor	10
T1	02
T2	05
T3	11
T4	22
**Regional extension: lymph node staging (cN)**	
N0	16
N1	11
N2	16
N3	7
**Differentiation (grading)**	
No grading (normal mucosa)	10
Well	18
Moderate	20
Poor	2

Each FFPE block was selected for analysis with ample tumour tissue area for each case of OSCC. The usual five-micron thick unstained sections were cut from the FFPE blocks and mounted on charged glass slides. Standard immunohistochemistry (IHC) staining procedure was performed to check the expression of the selected genes. Polyclonal antibody against human periostin (OSF-2/periostin, was procured from BioVendor Laboratory Medicine, Modrice, Czech Republic. Polyclonal Anti-CAV1 and both Mouse Monoclonal Anti TNC-c and Anti FSCN 1 Antibody obtained from Biogenex, Fremont, CA. Normal mucosal epithelium obtained from the third molar impacted surgery used as a positive control. Recommendations by the manufacturer were followed for optimum antibody dilution. A three-step indirect process was followed based on the streptavidin–biotin complex, with peroxidase conjugated streptavidin molecules and the brown colour due to 3′-diaminobenzidine substrate chromogen formed at the histological site of the target antigen. Haematoxylin was used as a counterstain.

A semi-quantitative method was used to score the sections of OSCC cases as described in earlier studies^15,16^. The individual sections were scored by inter-observers (Oral Histopathologists-SR and NM) to avoid bias. Sections were scored considering both the intensity and percentage of staining in the cells. For each antibody, a five tier scoring was done on scale of 0 to 4, where 0 was no staining, 1 in case of 25% (mild staining), 2 for 25–50% (medium staining), 3 for 50–75% (moderate staining) and 4 for ≥ 75% (strong staining)^17^. Further scoring was based on staining colour (reaction product) intensity as no staining, mild, medium, moderate and strong with scores of 0, 1, 2, 3, 4 respectively. The corresponding score for each sample in each slide for the four genes was calculated separately. Statistical analysis for the IHC data performed using GraphPad Prism 5 software. The categorical data compared to know the difference using the χ^2^ test. Fisher's exact test used to detect the association of a gene with OSCC. A nonparametric unpaired Mann–Whitney test used to compare the mean of two independent groups, e.g. healthy and cancer's stage and grade^18^. The p-value < 0.05 was considered significant for each statistical test^10^.

## Results

### Bioinformatics

#### Prediction of OSCC candidate genes

**Figure 1:**
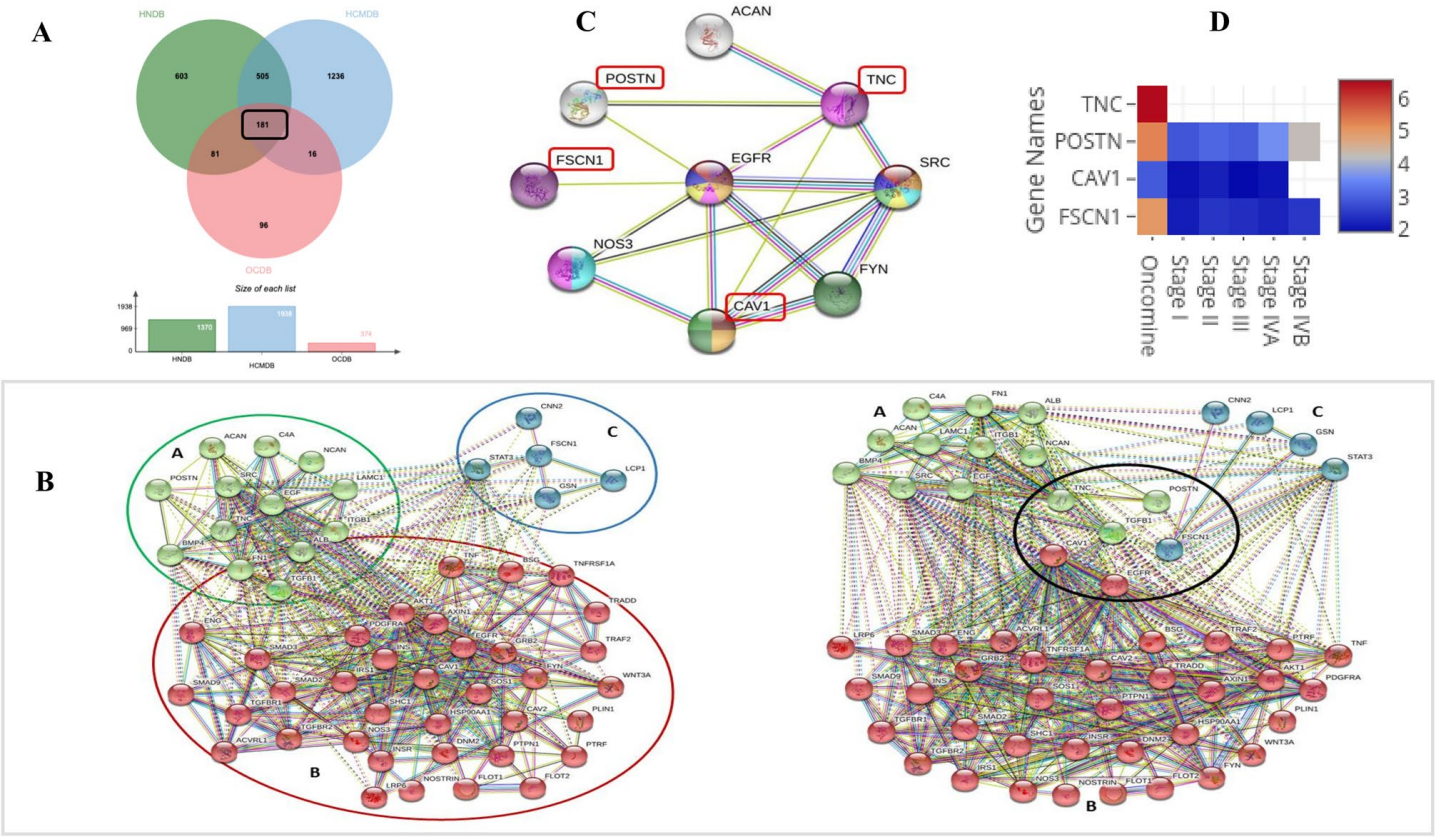
(**A**) Correlation among these database gene list revealed 181 common genes, metastatic in origin. (**B**) The cluster analysis of predicted 54 genes using STRING. The identified clusters are coloured in green (**A**), red (**B**) and blue (**C**). The solid and the dotted lines indicate connection within the same and different cluster respectively. Different colour indicates different type of interactions. b: Four genes of relevance in accordance to the interaction with TGF β and EGFR clustered together on basis of literature review. (**C**) The analysis of PPIs using STRING to identify interaction of candidate genes POSTN, TNC, CAV1 and FSCN1 in OSCC. (**D**) Heat map representing the average fold change compared to the normal sample from Oncomine (mRNA, OSCC) and Cancer RNA-Seq Nexus (RNA-Seq data, HNSCC).

Genes common among HCMDB (1938 metastatic genes), OrCaDB (374 genes) and HNdb database (1370 genes) were identified using jVenn (Fig. 1A) with the idea to identify known metastatic genes that are also reported to have an established role in oral cancer aetiology. A network was generated using the identified common genes using the STRINGdb with medium confidence level (0.7) for interactions. A cluster analysis was performed for metastatic genes around Tissue growth factor-β (TGF-β) and Epidermal growth factor receptor (EGFR) nodes, using K-means clustering method in STRINGdb. The strategy yielded a total of 54 genes within three clusters (referred to as clusters A, B and C) (Fig. 1B). The cluster A had 13 members, cluster B had 36 genes (maximum), and cluster C was very small with only 5 genes. The interaction via FN1, SRC and STAT3 nodes predicted four closely associated genes POSTN, TNC, CAV1 and FSCN1. A detailed literature survey carried amongst the genes from identified clusters revealed interesting link between these genes in particular. They were found to be directly interacting with TGF β and had literature linked to CAF’s and OSCC TME. These genes together did not present any relationship with the progression of OSCC in any of the databases. When initially reviewed for this study, it was interesting to see these genes had an individual role to play in OSCC pathogenesis. When linked together through PPI interaction showed evidence of a connected pathway for OSSC progression and thus can act as a panel of potential predictors (PPI enrichment p-value 0.027) (Fig. 1C). This provided a base for initiating a hypothesis for identification and assessment of candidate biomarkers in invasion and metastasis of OSCC.

#### Evidence in literature/databases for predicted candidate genes

The predicted genes were checked for their differential mRNA expression in oral cancer samples (OSCC/TSCC) in the Oncomine database (https://www.oncomine.org) and in the Expression Atlas (https://www.ebi.ac.uk/gxa/). The analysis showed that POSTN, TNC and FSCN1 are highly upregulated in OSCC with fold changes of 5.31, 6.55 and 5.05 respectively, whereas CAV1 showed a mild upregulation (fold change = 2.97) in Oncomine database (Tables 4, 5).

**Table 4:** The average fold change seen in candidate genes in Oncomine analysis.

	Fold change (Oncomine)
POSTN	5.31
TNC	6.55
CAV1	2.97
FSCN	5.05

**Table 5:** Detailed panel of candidate OSCC genes.

Gene	DATA set	Site	No. of normal sample	No. of cancer sample	Fold change	P value
POSTN	Estilo head-neck	TSCC vs. normal	31	26	6.089	6.29E−10
POSTN	Talbot lung	TSCC vs. normal	31	26	4.525	1.24E−09
TNC	Talbot lung	TSCC vs. normal	31	26	7.202	7.85E−16
TNC	Peng head-neck	OSCC vs. normal	57	22	4.909	9.46E−26
TNC	Estilo head-neck	TSCC vs. normal	31	26	8.627	8.79E−14
TNC	Pyeon multi-cancer	Tongue carcinoma vs. normal	15	9	5.459	2.28E−08
CAV1	Pyeon multi-cancer	Tongue carcinoma vs. normal	15	9	3.727	3.91E−06
CAV1	Estilo head-neck	TSCC vs. normal	31	26	2.203	4.05E−05
FSCN1	Estilo head-neck	TSCC vs. normal	31	26	7.417	3.49E−14
FSCN1	Talbot lung	TSCC vs. normal	31	26	7.084	2.26E−16
FSCN1	Peng head-neck	OSCC vs. normal	57	22	2.966	1.93E−22
FSCN1	Kuriakose head-neck	TSCC vs. normal	22	3	2.713	1.85E−04

The mRNA expression status (OSCC/TSCC vs normal) of a gene as reported in the Oncomine database).

The analysis of mRNA sequencing data from Cancer RNA-Seq Nexus (CRN) database revealed these genes in all stages showed a fold change higher than 2, except for CAV1 in Stage III and Stage IV A (Table 6). A comparative heat map generated using Oncomine and CRN data showed TNC, POSTN and FSCN1 are significantly upregulated whereas CAV1 is moderately upregulated. TNC and CAV1 had the highest and lowest upregulation respectively in Oncomine data. The CRN does not have data for TNC. The POSTN and FSCN1 data showed increasing expression level as clinical stage progressed, whereas the CAV1 showed constant upregulation through the clinical stages (Fig. 1D).

**Table 6:** Detailed panel of candidate OSCC genes.

Genes	Stage I	Stage II	Stage III	Stage IVA	Stage IVB
POSTN	2.91	3.12	3.01	3.63	4.32
CAV1	2.05	2.24	1.96	2.10	–
FSCN1	2.20	2.50	2.42	2.29	2.53

Fold change of RNA-Seq data (OSCC/TSCC) of genes as reported in the Cancer RNA-Seq Nexus database categorised as per staging.

### Proteomics

**Figure 2:**
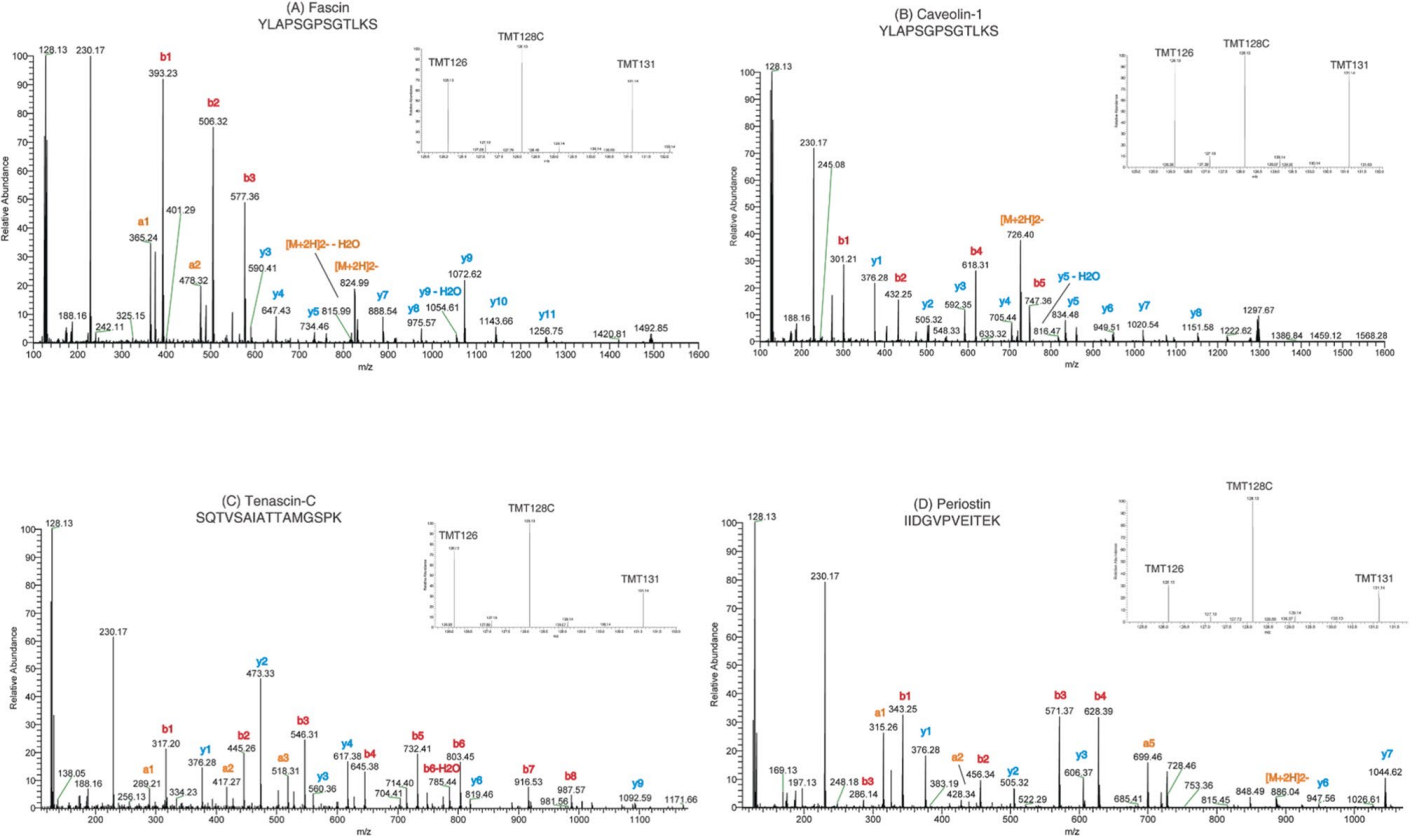
Representative MS/MS spectra of identified peptides from Periostin (POSTN); Tenascin c (TNC); Caveolin 1 (CAV1) and Fascin 1 (FSCN1). The MS spectra in the inset provides the relative abundance with Tandem Mass Tags (TMTs) of the representative peptides.

**Figure 3:**
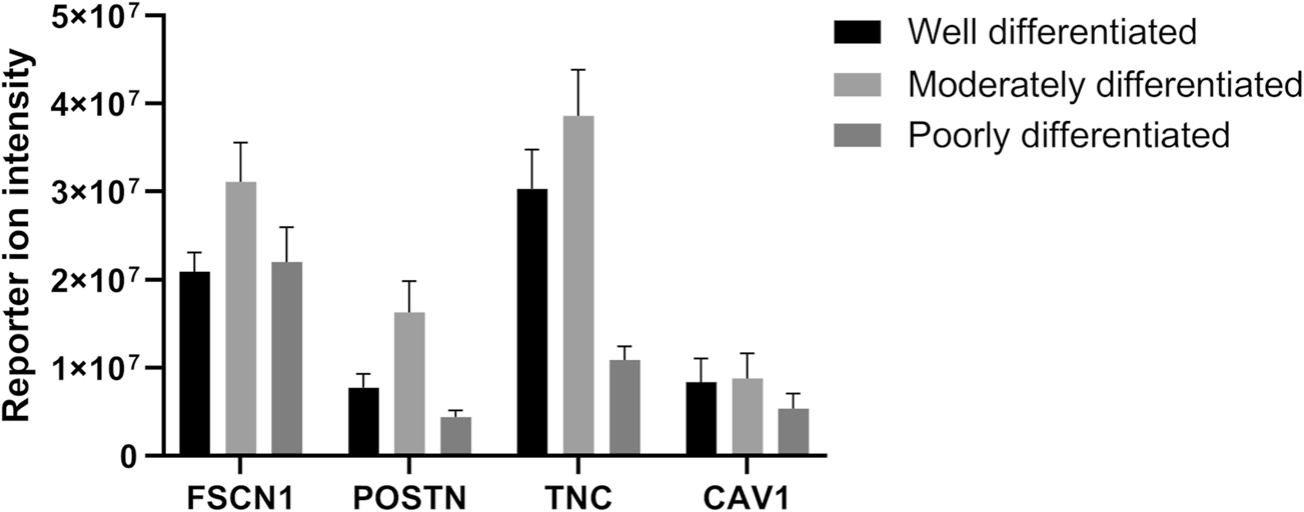
Graphical representation of fold change difference when in comparison to different grades in proteomic analysis.

Proteomic profiling using PCT and TMT-based mass spectrometry analysis was performed. The cohort cases majorly comprised of habit history of chewing tobacco in 11 of the 15 participants, whereas only one patient had a history of using smoking and chewing tobacco along with alcohol habit. Majority cases belonged to the gingivobuccal complex (GBC) site and Stage IV as per clinical staging. The cases of all assorted grades taking five each from various histopathological differentiation. In our spectrum, the base peak of the four candidate genes showed the greatest relative abundance (100%) along with other peaks corresponding to ion fragments. The proteins identified IIDGVPVEITEK (POSTN), SQTVSAIATTAMGSPK (TNC), YLAPSGPSGTLKS (CAV1), YLAPSGPSGTLKS (FSCN1) (Fig. 2). Analysis of the fold change pattern revealed increased expression of POSTN, TNC, CAV1, and FSCN1 in moderately differentiated OSCC indicative of their role in invasion and metastasis. (Table 7). In the graphical representation of the differential expression related to the genes, TNC showed the highest fold change difference when moderately differentiated OSCC samples were compared to poorly differentiated cases. POSTN too had enumerable fold change difference when in comparison to different grades (Fig. 3). When p-values were calculated, TNC had a highly significant value in well vs poorly differentiated cases (p < 0.0001). Further, POSTN also showed a significant difference when well-differentiated cases were compared to poorly-differentiated cases (Table 8).

**Table 7:** Fold change expression of POSTN, TNC, CAV1, FSCN1 in various grades.

Gene symbol	Well differentiated vs. moderately differentiated	Moderately differentiated vs. poorly differentiated	Well differentiated vs. poorly differentiated
FSCN1	0.65	1.48	0.96
POSTN	0.49	3.33	1.65
TNC	0.78	3.45	2.71
CAV1	0.94	1.67	1.57

**Table 8:** p-values of genes in various comparative grades.

Gene symbol	P value (well differentiated vs. moderately differentiated)	P value (moderately differentiated vs. poorly differentiated)	P value (well differentiated vs. poorly differentiated)
FSCN1	0.01	0.02	0.65
POSTN	0.004	0.0006	0.01
TNC	0.05	0.00005	0.0001
CAV1	0.84	0.08	0.10

### Immunohistochemistry

**Figure 4:**
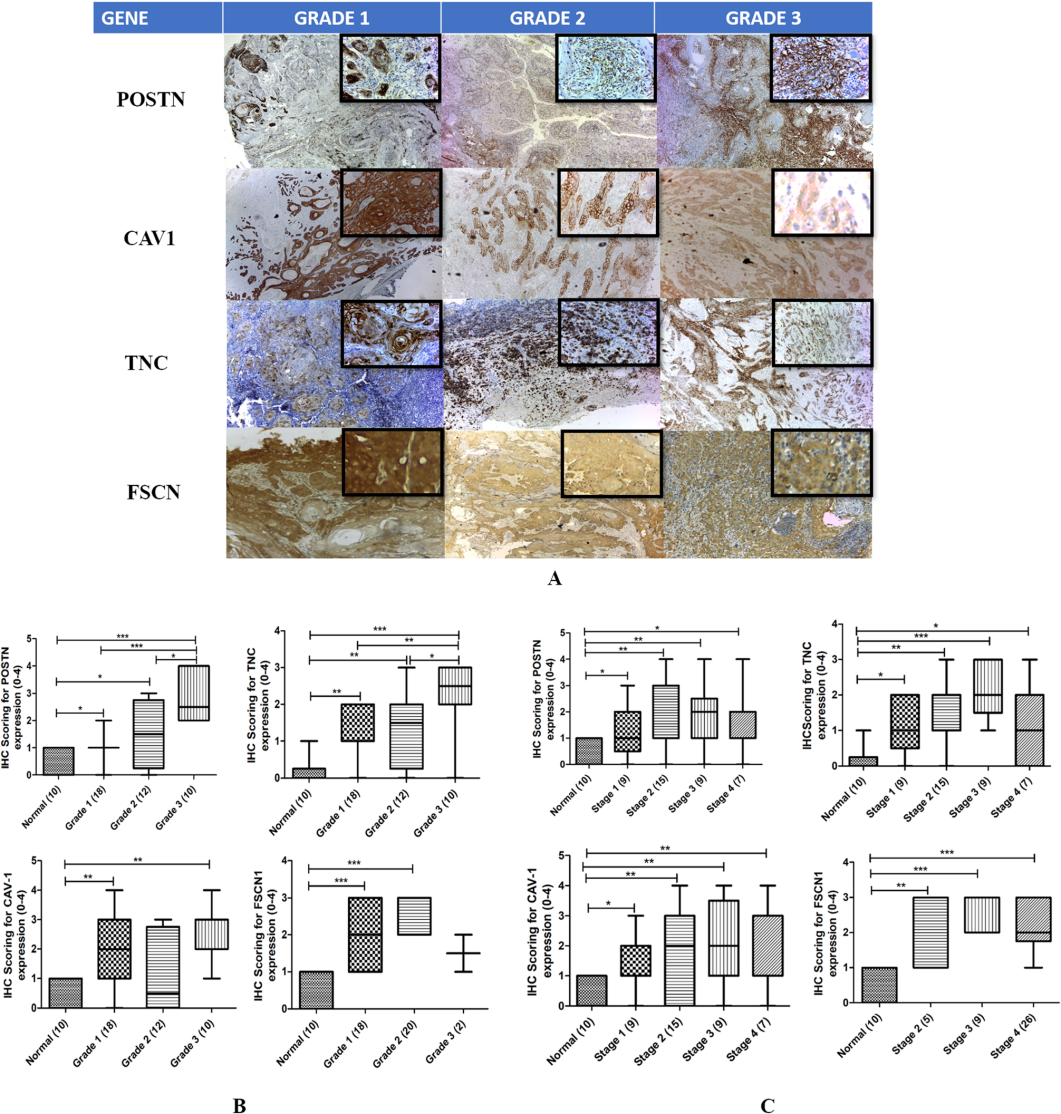
(**A**) Representative figure of immunohistochemically stained sections of OSCC for the candidate genes POSTN, TNC, CAV1, FSCN 1 according to the grades. The IHC results clearly indicate that the expression gets upregulated with the advancing cancer microenvironment with cytoplasmic staining. (**B**) Box plot showing the Grade wise expression of the genes on immunohistochemically stained sections of OSCC samples. (**C**) Box plots showing Stage wise expression of the genes on immunohistochemically stained sections of Normal and OSCC samples.

The expression of POSTN, TNC, CAV1 and FSCN1 was localized predominantly in the cytoplasm of the tumour cells. Though in TNC and CAV1, immunoreactivity was observed both in membrane and intra-cytoplasmic of some sections. The grade wise staining pattern is described in Fig. 4A. Amongst the 40 OSCC patients, 25 participants were smokeless tobacco (ST) user and 27 of them had tumour located at gingivobuccal sulcus (GBC) in the POSTN, TNC and CAV1 group. For FSCN1 study group, all the participants were smokeless tobacco (ST) users and had tumour located at gingivobuccal sulcus (GBC) except the controls. The statistical analysis by Mann–Whitney U test for grade wise analysis (Fig. 4B) revealed the genes POSTN, TNC, FSCN1 were differentially upregulated whereas CAV1 showed no significant change. For POSTN and TNC, significant difference was observed between normal vs. grade 1, normal vs. grade 2 and normal vs. grade 3 comparisons. A significant difference was observed amongst the grades (grade 1 vs grade 2 vs grade 3) related to POSTN and TNC. CAV1 had a significant difference in mean expression between normal and grade 1 and grade 3. In FSCN1, statistically significant difference was observed between normal and grade 1 and grade 2. The POSTN, TNC and CAV1 mean expression was significantly different between normal vs stage 1, normal vs. stage 2, normal vs. stage 3 and normal vs. stage 4 (Fig. 4C) whereas the results were insignificant when compared between all the stages. In case of FSCN 1, significant difference was found between normal and stage 2, stage 3 and stage 4. All the four genes in both grade and stage wise comparison showed significant differences in expression from normal to OSCC samples.

### Hypothesis

TGF-β1 signalling known to induce myofibroblastic differentiation depending on the expression of ECM proteins^19,20^. Under the hypoxic environment, CAF’s and TGF β reciprocally induce invasion^21^. TGF-β3, in particular, has been found to trigger the induction of POSTN in CAF’s and induces production of stromal POSTN^22^ and in turn CAF's signalled by POSTN leads to the secretion of ECM protein^23^. Further POSTN performs as a ligand for integrin's αvβ3 and αvβ5, promoting activation of multiple pathways like PI3kinase-Akt pathway leading to increased tumour cell invasion^24,25^. While regulating the EMT, POSTN plays a role in cancer stemness via interacting with protein tyrosine kinase 7 (PTK7) and propagates the cancer stem cell (CSC)-like phenotype via PTK7-Wnt/β-Catenin signalling^26^. CAF's are able enhancer of tumour invasion through their secretion of TNC-c, TNC-w, HGF, and MMPs along with TGF-β^27^. POSTN was also found to incorporate TNC into the TME and promote stiffening of ECM in the cancer niche, thereby releasing the active form of TGF-β1 regulating cancer cell proliferation via ERK signalling pathway^28^. Further downregulation of CAV1 in TME promotes adjacent normal fibroblasts into CAF's phenotype and stimulates Rho- and force-dependent contraction, matrix alignment^21^. This TME stiffening through regulation of p190RhoGAP favours directional migration and invasiveness of carcinoma cells^10^. This illuminates a link between CAV1 and FSCN1 in facilitating of cell migration, invasion and metastasis via Src/FAK pathway and filopodia formation^29^. CAV1, delivered by cancer cell-derived exosomes and its accumulation in OSCC TME promoted EMT and trans-differentiation of fibroblasts junto CAF's^23^.

**Figure 5:**
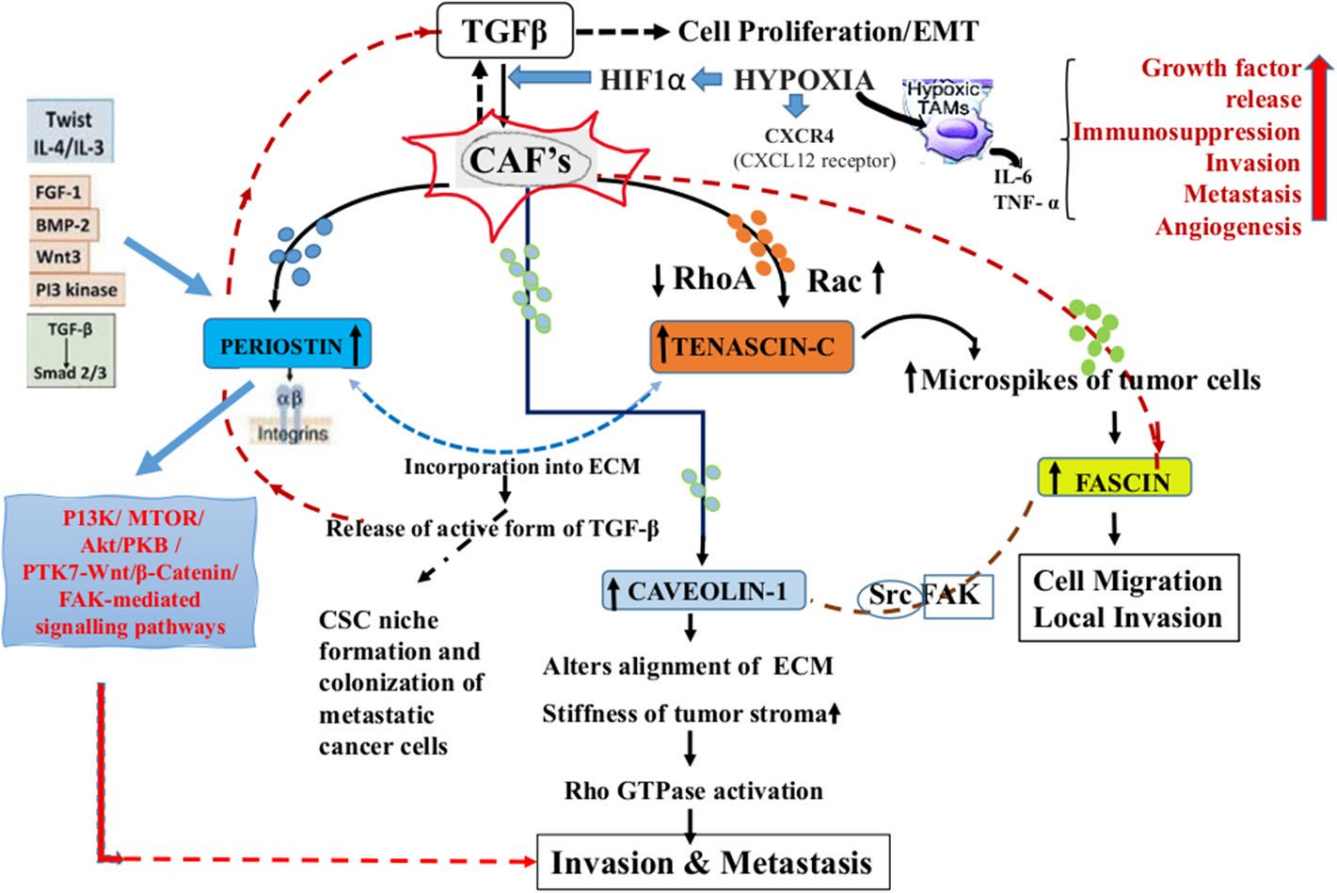
Proposed role of POSTN, TNC, CAV1 and FSCN1, their interaction with each other and CAF’s in OSCC tumour-microenvironment.

Thereby, taking the above thorough literature survey into account there is an evident link in their pathways related to invasion, progression and metastasis, they have never been studied earlier as a panel for candidate biomarkers. We propose a mechanism/pathway (Fig. 5) to enumerate the occurrence of the four genes POSTN, TNC, CAV1and FSCN1 in OSCC TME via the role of CAF's and TGF-β, as a panel of candidate biomarkers for OSCC.

## Discussion

India, a country with a population of about 1.3 billion, presents lip and oral cavity cancer as prevalent cancer amongst men in the global map. According to the World Health Organization (WHO), in 2015, cancer was the first or second leading cause of death before age 70 years in as many as 91 countries. The GLOBOCAN 2018 database for 185 countries and 36 cancers states lip and oral cavity cancer cluster in certain high-risk regions like India and are more frequent in males^30^. According to Global Cancer Observatory (https://gco.iarc.fr/tomorrow, accessed 20-01-2021) incidence and mortality in world population for both male and female is predicted 553 K and 263 k (47.55%) respectively by 2040. While the incidence and mortality in Male only is 382 K and 182 K (46.64%) respectively. So according to this data male are on higher risk of incidence (70.34%) and mortality (69.2%) comparative to female incidence (30.92%) mortality (30.87%).

ST use in adults Its already reported that smokeless tobacco (ST) use though prevalent in 127 countries worldwide; the highest rates of consumption and risk estimates for oral cancer is in South and Southeast Asia^31^. Global Adult Tobacco Survey India 2016–2017 (GATS 2, https://www.who.int/tobacco/surveillance/survey/gats/GATS_India_2016-17_FactSheet.pdf?ua=1) attributed ST, alcohol use and smoking as leading risk factors for lip and oral cavity cancer in India.

Over the years, the need for finding a tumour biomarker for the prognosis and better treatment protocol for OSCC patients has been tenacious. Literature survey related to OSCC did yield few relevant studies which have attempted to find a candidate biomarker in the field of OSCC, but the haunt is still on^7,32^. Hypothesis proposed in our study establishes an interface between pathways related to invasion and metastasis in OSCC TME via the role of CAF's and TGF-β and the four genes POSTN, TNC (both extracellular matrix proteins), CAV1 (a scaffolding protein) and FSCN1 (organization of actin filament bundles). Similar to this study, an integrated bioinformatics analysis approach has already been reported to predict candidate genes related to OSCC in the past^10^. It is pertinent to note that the lack of targeted therapies is an imperative reason behind low survival rate in HNSCC and in particular OSCC. Our study attempts to provide a comprehensive perspective to understand underlying mechanisms in OSCC and pivotal pathways that may be exploited to develop targeted therapeutics. Based on our hypothesis, we co-related the expression and interface between these genes with evidence collected from literature review. Kikuchi et al., initiated an association between CAF's and POSTN, in 2008 using in-situ hybridisation (ISH)^33^. POSTN secreted by CAFs, promotes activation of the PI3kinase-Akt pathway established in 2015^24^. CAF-derived POSTN plays a role in cancer stemness via interacting with Protein tyrosine kinase 7 (PTK7) in HNSCC^26^. Even, Tn-C/TNC in OSCC, transpires as different isoforms generated by alternative splicing and de novo glycosylation^34^. For understanding the association of CAF's and CAV1, a study in 2010 reported downregulation of CAV1 in TME promoted adjacent normal fibroblasts into CAF's phenotype^5^. Further, CAV1 stimulates Rho- and force-dependent contraction, matrix alignment, and TME stiffening through regulation of p190RhoGAP favouring directional migration and invasiveness of carcinoma cells in vitro. Extracting the link between CAV1 and FSCN1 revealed facilitation of cell migration, invasion and metastasis^29^. Another study found that TGF β1, Epidermal growth factor (EGF) and Interleukin 1β (IL 1β) significantly stimulated FSCN1 expression and even suggested that RhoA and Nuclear factor kappa B (NF κB) signals were involved in the same.

After considering all pieces of evidence, we further initiated proteomic profiling of these four genes POSTN, TNC, CAV1 and FSCN1 which have shown to be part of the modulation process of TME via CAF’s and TGFβ pathways. A SELDI-TOF protein chip system used to screen proteins in saliva from pre- and post-treatment OSCC samples, too displayed an altered pattern of proteins^35^. Using the proteomics data and bioinformatics results, the prioritization index of biomarker candidates for IHC on tissue revealed POSTN as a top candidate^36^. Various studies found upregulation of TNC and validated with IHC^37–39^. Using proteomics and bioinformatics techniques, CAV1 has been identified as a major network-centric protein between gastric cancer-associated fibroblasts (GCAFs) and their corresponding inflammation-associated fibroblasts (GIAFs) which was later validated using IHC^40^. Very few studies were found to be assessing FSCN1 role in malignancy using proteomic profiling and validation by immunohistochemistry further revealing its role as a specific target^41^. On literature search for proteomic profiling of POSTN, TNC, CAV1 and FSCN1 in OSCC or oral cancer, we could not find any related studies reported till date.

We followed the protocol as suggested in the study using FFPE tissue samples in a PCT and TMT-based mass spectrometry analysis approach, and bioinformatics results for isolation of candidate biomarkers^36^. Though the data was consistent with several other types of cancers, we could not find any relevant comparative analysing data with OSCC for our panel of candidate biomarkers. Considering this, we shall report as one of the front runners in proteomic profiling data for our candidate biomarkers POSTN, TNC, CAV1, FSCN1.

Multiple studies using IHC conveys, POSTN to be upregulated at the invasive front in both tumour epithelia and the surrounding matricellular space^42–44^. When studied in OSCC/HNSCC, the expression of POSTN in the epithelium is associated with a more aggressive tumour phenotype in OSCC, as was determined by the mRNA and IHC expression^45^. Also, upregulation of POSTN gene expression and establishment of its role in tumour lymphangiogenesis, making it evident that this can be used as a potential biomarker for OSCC^46,47^. In a study in 2006, POSTN expression was well correlated with the invasion pattern and metastasis. TNC upregulation at the invasive tumour front has already been associated with poor clinical outcome suggesting its role in metastatic progression^48^. Taking various cancers into account, CAV1 was found to be downregulated in some while increased expression seen in a few others, indicative of a biphasic nature of CAV1^49^. In a total of 26 IHC studies of 5 prevalent human carcinomas when identified for meta-analysis found FSCN1 was associated with increased risk of mortality for breast and oesophageal carcinomas. A number of studies have demonstrated that FSCN 1 might be playing a role in malignancy and also have suggested a significant correlation between the expression of FSCN1 with local lymph node metastasis^15^. Role of FSCN1 as a prognostic biomarker for OSCC cases was first concluded in 2007^50^. To better explain the role of this protein, several studies have investigated its function, including a study on two OSCC cell lines concluded that FSCN1 expression might have an essential role in the regulation and development of OSCC that acts through epithelial-mesenchymal transition (EMT) and changes in E-cadherin and β-catenin expressions^51^. Actin components such as microspikes were found to be thicker and longer and showed the formation of more filopodia and lamellipodia, depicting the role of FSCN in disrupting the cell–cell contact and was instrumental in the progression of primary OSCC tumor^52^. FSCN1 over-expression in lymph nodes were significantly associated with clinico-histopathological parameters^15^.

Considering IHC as a robust validation methodology, we validated the results of our bioinformatics analysis and proteomic profiling of our panel of candidate genes using IHC. In our study, the expression of POSTN, TNC, CAV1 and FSCN1 found to be localized predominantly in the cytoplasm of the tumour cells. It is worth to reiterate that these genes have shown significant expression differences from normal to OSCC samples (all stages and grades). Thereby the upregulation from normal to different grades and stages does indicate the expression of protein through invasion and progression, in due course preceding to metastasis.

For taking up IHC validation, we included samples of all grades and stages in group 1 maintaining heterogeneity. In group 2, no samples for Stage 1 and only 2 samples in Grade III were included because in this group, lymph node assessment and survival analysis was included for few patients having follow up data We do consider this a as a limitation of our study.

About CAV1’s role in OSCC, overexpression in the cytoplasm of OSCC and its upregulation was in sync with tumour progression. Increased CAV1 expression is seen in the stepwise carcinogenesis from normal to primary OSCC. In contrast, decrease in expression seen between the grades and stages or primary OSCC to metastatic OSCC indicating at its biphasic functions^49^. This may owe to the theory of a reverse Warburg metabolism in OSCC that reduced CAV1 can lead to an increase in oxidative stress promoting metastasis^53^. The data in our study matched this logic as it showed upregulated expression for normal vs grades and stages in case of CAV1.

## Conclusion

The current study has used an integrated approach utilising bioinformatics, proteomics and immunohistochemistry for identification of candidate biomarkers and their validation. Identification of four potential candidate genes and a hypothesis was generated for their plausible role in invasion and metastasis. The experimental validation of predicted genes through proteomic profiling using PCT and TMT-based mass spectrometry and immunohistochemical analysis indicated the robustness of the current approach. We anticipate that the identified genes will aid into our understanding of the molecular mechanisms underlying the invasion and metastasis and be of assistance in identifying novel targeted therapeutics (Supplementary Information).

## Supplementary Information

Supplementary Information.

## References

[1] https://gco.iarc.fr/tomorrow/en/dataviz/isotype?cancers=1&single_unit=50000&sexes=0

[2] Stuelten CH, Parent CA, Montell DJ (2018). Cell motility in cancer invasion and metastasis: Insights from simple model organisms. Nat. Rev. Cancer.

[3] Bhattacharya R, Panda CK, Nandi S, Mukhopadhyay A (2018). An insight into metastasis: Random or evolving paradigms?. Pathol. Res. Pract..

[4] Jiang WG (2015). Tissue invasion and metastasis: Molecular, biological and clinical perspectives. Semin. Cancer Biol..

[5] Martinez-Outschoorn UE (2010). Tumor cells induce the cancer associated fibroblast phenotype via caveolin-1 degradation: Implications for breast cancer and DCIS therapy with autophagy inhibitors. Cell Cycle.

[6] Siriwardena S, Tsunematsu T, Qi G, Ishimaru N, Kudo Y (2018). Invasion-related factors as potential diagnostic and therapeutic targets in oral squamous cell carcinoma—A review. Int. J. Mol. Sci..

[7] Rivera C, Oliveira AK, Costa RAP, De Rossi T, Paes Leme AF (2017). Prognostic biomarkers in oral squamous cell carcinoma: A systematic review. Oral Oncol..

[8] Almangush A (2017). Prognostic biomarkers for oral tongue squamous cell carcinoma: A systematic review and meta-analysis. Br. J. Cancer.

[9] Rai V, Mukherjee R, Ghosh AK, Routray A, Chakraborty C (2018). "Omics" in oral cancer: New approaches for biomarker discovery. Arch. Oral Biol..

[10] Kumar R, Samal SK, Routray S, Dash R, Dixit A (2017). Identification of oral cancer related candidate genes by integrating protein-protein interactions, gene ontology, pathway analysis and immunohistochemistry. Sci. Rep..

[11] Bardou P, Mariette J, Escudie F, Djemiel C, Klopp C (2014). jvenn: An interactive Venn diagram viewer. BMC Bioinform..

[12] Zhu Y (2019). High-throughput proteomic analysis of FFPE tissue samples facilitates tumor stratification. Mol. Oncol..

[13] Shao S (2016). Reproducible tissue homogenization and protein extraction for quantitative proteomics using micropestle-assisted pressure-cycling technology. J. Proteome Res..

[14] Guo T (2015). Rapid mass spectrometric conversion of tissue biopsy samples into permanent quantitative digital proteome maps. Nat. Med..

[15] Routray S, Kheur S, Chougule HM, Mohanty N, Dash R (2017). Establishing Fascin over-expression as a strategic regulator of neoplastic aggression and lymph node metastasis in oral squamous cell carcinoma tumor microenvironment. Ann. Diagn. Pathol..

[16] Samal SK, Routray S, Veeramachaneni GK, Dash R, Botlagunta M (2015). Ketorolac salt is a newly discovered DDX3 inhibitor to treat oral cancer. Sci. Rep..

[17] McDonald JW, Pilgram TK (1999). Nuclear expression of p53, p21 and cyclin D1 is increased in bronchioloalveolar carcinoma. Histopathology.

[18] Meyerholz DK, Beck AP (2018). Principles and approaches for reproducible scoring of tissue stains in research. Lab. Investig..

[19] Gonda TA, Varro A, Wang TC, Tycko B (2010). Molecular biology of cancer-associated fibroblasts: Can these cells be targeted in anti-cancer therapy?. Semin. Cell Dev. Biol..

[20] Bhattacharya M (2002). Beta-arrestins regulate a Ral-GDS Ral effector pathway that mediates cytoskeletal reorganization. Nat. Cell Biol..

[21] Routray S (2014). Caveolin-1 in oral squamous cell carcinoma microenvironment: An overview. Tumour Biol..

[22] Qin X (2016). TGFbeta3-mediated induction of Periostin facilitates head and neck cancer growth and is associated with metastasis. Sci. Rep..

[23] Augsten M (2014). Cancer-associated fibroblasts as another polarized cell type of the tumor microenvironment. Front. Oncol..

[24] Routray, A. & Rahman, J. In *Encyclopedia of Signaling Molecules* (ed Choi, S.) 244–244 (Springer International Publishing, 2018).

[25] Underwood TJ (2015). Cancer-associated fibroblasts predict poor outcome and promote periostin-dependent invasion in oesophageal adenocarcinoma. J. Pathol..

[26] Yu B (2018). Periostin secreted by cancer-associated fibroblasts promotes cancer stemness in head and neck cancer by activating protein tyrosine kinase 7. Cell Death Dis..

[27] Shimoda M, Mellody KT, Orimo A (2010). Carcinoma-associated fibroblasts are a rate-limiting determinant for tumour progression. Semin. Cell Dev. Biol..

[28] Kikuchi Y (2014). The niche component periostin is produced by cancer-associated fibroblasts, supporting growth of gastric cancer through ERK activation. Am. J. Pathol..

[29] Goetz JG (2011). Biomechanical remodeling of the microenvironment by stromal caveolin-1 favors tumor invasion and metastasis. Cell.

[30] Bray F (2018). Global cancer statistics 2018: GLOBOCAN estimates of incidence and mortality worldwide for 36 cancers in 185 countries. CA Cancer J. Clin..

[31] Siddiqi K (2020). Global burden of disease due to smokeless tobacco consumption in adults: An updated analysis of data from 127 countries. BMC Med..

[32] Blatt S (2017). Biomarkers in diagnosis and therapy of oral squamous cell carcinoma: A review of the literature. J. Craniomaxillofac. Surg..

[33] Kikuchi Y (2008). Periostin is expressed in pericryptal fibroblasts and cancer-associated fibroblasts in the colon. J. Histochem. Cytochem..

[34] Berndt A, Richter P, Kosmehl H, Franz M (2015). Tenascin-C and carcinoma cell invasion in oral and urinary bladder cancer. Cell Adh. Migr..

[35] Shintani S, Hamakawa H, Ueyama Y, Hatori M, Toyoshima T (2010). Identification of a truncated cystatin SA-I as a saliva biomarker for oral squamous cell carcinoma using the SELDI ProteinChip platform. Int. J. Oral Maxillofac. Surg..

[36] Lai X, Chen S (2015). Identification of novel biomarker candidates for immunohistochemical diagnosis to distinguish low-grade chondrosarcoma from enchondroma. Proteomics.

[37] Rupp T (2016). Tenascin-C orchestrates glioblastoma angiogenesis by modulation of pro- and anti-angiogenic signaling. Cell Rep..

[38] Gocheva V (2017). Quantitative proteomics identify Tenascin-C as a promoter of lung cancer progression and contributor to a signature prognostic of patient survival. Proc. Natl. Acad. Sci. USA..

[39] Zheng J (2018). Extracellular matrix proteins and carcinoembryonic antigen-related cell adhesion molecules characterize pancreatic duct fluid exosomes in patients with pancreatic cancer. HPB.

[40] Shen XJ (2015). Caveolin-1 is a modulator of fibroblast activation and a potential biomarker for gastric cancer. Int. J. Biol. Sci..

[41] Poli G (2015). 2D-DIGE proteomic analysis identifies new potential therapeutic targets for adrenocortical carcinoma. Oncotarget.

[42] Soltermann A (2012). Epithelial-mesenchymal transition in non-small cell lung cancer. Pathologe.

[43] Gunia S (2013). Periostin expression correlates with pT-stage, grading and tumour size, and independently predicts cancer-specific survival in surgically treated penile squamous cell carcinomas. J. Clin. Pathol..

[44] Wu SQ (2013). Silencing of periostin inhibits nicotine-mediated tumor cell growth and epithelial-mesenchymal transition in lung cancer cells. Mol. Med. Rep..

[45] Luo JH, Zhou J, Gao Y (2013). Correlation between periostin and SNCG and esophageal cancer invasion, infiltration and apoptosis. Asian Pac. J. Trop. Med..

[46] Siriwardena BS (2006). Periostin is frequently overexpressed and enhances invasion and angiogenesis in oral cancer. Br. J. Cancer.

[47] Choi P (2008). Examination of oral cancer biomarkers by tissue microarray analysis. Arch. Otolaryngol. Head Neck Surg..

[48] Lowy CM, Oskarsson T (2015). Tenascin C in metastasis: A view from the invasive front. Cell Adh. Migr..

[49] Hung KF (2003). The biphasic differential expression of the cellular membrane protein, caveolin-1, in oral carcinogenesis. J. Oral Pathol. Med..

[50] Lee TK (2007). Fascin over-expression is associated with aggressiveness of oral squamous cell carcinoma. Cancer Lett..

[51] Chen SF (2007). Expression of fascin in oral and oropharyngeal squamous cell carcinomas has prognostic significance—A tissue microarray study of 129 cases. Histopathology.

[52] Alam H (2012). Fascin overexpression promotes neoplastic progression in oral squamous cell carcinoma. BMC Cancer.

[53] Routray S, Sunkavali A, Bari KA (2014). Carcinoma-associated fibroblasts, its implication in head and neck squamous cell carcinoma: A mini review. Oral Dis..

