# Supplementary material for: An integrated approach for identification of a panel of candidate genes arbitrated for invasion and metastasis in oral squamous cell carcinoma
Source: Sci Rep. 2021 Mar 18;11:6208. doi: 10.1038/s41598-021-85729-x (PMC7973753; doi:10.1038/s41598-021-85729-x)
Supplement: Supplementary file 1 — Supplementary Information. [file 41598_2021_85729_MOESM1_ESM.docx]

Supplementary data:

**Table 1**: Biological Process (GO) involving the genes described in STRING analysis.

| **Pathway ID** | **Pathway description** | **Count in gene set** |
| --- | --- | --- |
| GO:0022612 | Gland morphogenesis | 4 |
| GO:0048010 | Vascular endothelial growth factor receptor signalling pathway | 4 |
| GO:0086098 | Angiotensin-activated signalling pathway involved in heart process | 2 |
| GO:2001236 | Regulation of extrinsic apoptotic signalling pathway | 4 |
| GO:0050730 | Regulation of peptidyl-tyrosine phosphorylation | 4 |
| GO:0031295 | T cell co-stimulation | 3 |
| GO:0007169 | Transmembrane receptor protein tyrosine kinase signalling pathway | 5 |
| GO:0003057 | Regulation of the force of heart contraction by chemical signal | 2 |
| GO:0009611 | Response to wounding | 5 |
| GO:0016477 | Cell migration | 5 |
| GO:0003018 | Vascular process in circulatory system | 3 |
| GO:0048771 | Tissue remodelling | 3 |
| GO:2001237 | Negative regulation of extrinsic apoptotic signalling pathway | 3 |
| GO:0051674 | Localization of cell | 5 |
| GO:0036120 | Cellular response to platelet-derived growth factor stimulus | 2 |
| GO:0048608 | Reproductive structure development | 4 |
| GO:0048732 | Gland development | 4 |
| GO:0061458 | Reproductive system development | 4 |
| GO:0050865 | Regulation of cell activation | 4 |
| GO:2000811 | Negative regulation of anoikis | 2 |
| GO:0009968 | Negative regulation of signal transduction | 5 |
| GO:0007596 | Blood coagulation | 4 |
| GO:0008543 | Fibroblast growth factor receptor signalling pathway | 3 |
| GO:0018108 | Peptidyl-tyrosine phosphorylation | 3 |
| GO:0042522 | Regulation of tyrosine phosphorylation of Stat5 protein | 2 |
| GO:0061564 | Axon development | 4 |
| GO:0071417 | Cellular response to organonitrogen compound | 4 |
| GO:0032870 | Cellular response to hormone stimulus | 4 |
| GO:0071560 | Cellular response to transforming growth factor beta stimulus | 3 |
| GO:0044344 | Cellular response to fibroblast growth factor stimulus | 3 |
| GO:0046777 | Protein autophosphorylation | 3 |
| GO:0070372 | Regulation of ERK1 and ERK2 cascade | 3 |
| GO:0007173 | Epidermal growth factor receptor signalling pathway | 3 |
| **Pathway ID** | **Pathway description** | **Count in gene set** |
| GO:0043267 | Negative regulation of potassium ion transport | 2 |
| GO:0046209 | Nitric oxide metabolic process | 2 |
| GO:0038093 | Fc receptor signalling pathway | 3 |
| GO:0080134 | Regulation of response to stress | 5 |
| GO:0042060 | Wound healing | 4 |
| GO:0034405 | Response to fluid shear stress | 2 |
| GO:0050900 | Leukocyte migration | 3 |
| GO:0032570 | Response to progesterone | 2 |
| GO:0042127 | Regulation of cell proliferation | 5 |
| GO:0038083 | Peptidyl-tyrosine autophosphorylation | 2 |
| GO:0051017 | Actin filament bundle assembly | 2 |
| GO:0050999 | Regulation of nitric-oxide synthase activity | 2 |
| GO:0009888 | Tissue development | 5 |
| GO:0006468 | Protein phosphorylation | 4 |
| GO:0008015 | Blood circulation | 3 |
| GO:0044403 | Symbiosis, encompassing mutualism through parasitism | 4 |
| GO:0048011 | Neurotrophin TRK receptor signalling pathway | 3 |
| GO:0060443 | Mammary gland morphogenesis | 2 |
| GO:1901701 | Cellular response to oxygen-containing compound | 4 |
| GO:0045428 | Regulation of nitric oxide biosynthetic process | 2 |
| GO:0043066 | Negative regulation of apoptotic process | 4 |
| GO:0071375 | Cellular response to peptide hormone stimulus | 3 |
| GO:0003015 | Heart process | 2 |
| GO:1901888 | Regulation of cell junction assembly | 2 |
| GO:0030900 | Forebrain development | 3 |
| GO:0035556 | Intracellular signal transduction | 5 |
| GO:0071407 | Cellular response to organic cyclic compound | 3 |
| GO:0007155 | Cell adhesion | 4 |
| GO:0030198 | Extracellular matrix organization | 3 |
| GO:0045937 | Positive regulation of phosphate metabolic process | 4 |
| GO:0050880 | Regulation of blood vessel size | 2 |
| GO:0071396 | Cellular response to lipid | 3 |

**Table 2:** Cellular Component (GO) in STRING analysis

| **Pathway ID** | **Pathway description** | **Count in gene set** |
| --- | --- | --- |
| GO:0005901 | Caveola | 3 |
| GO:0045121 | Membrane raft | 4 |
| GO:0044431 | Golgi apparatus part | 5 |
| GO:0005794 | Golgi apparatus | 5 |
| GO:0030139 | Endocytic vesicle | 3 |
| GO:0005768 | Endosome | 4 |

| **Pathway ID** | **Pathway description** | **Count in gene set** |
| --- | --- | --- |
| 04510 | Focal adhesion | 5 |
| 04520 | Adherens junction | 3 |
| 04915 | Estrogen signalling pathway | 3 |
| 04611 | Platelet activation | 3 |
| 04921 | Oxytocin signalling pathway | 3 |
| 05206 | MicroRNAs in cancer | 3 |
| 04144 | Endocytosis | 3 |
| 05205 | Proteoglycans in cancer | 3 |
| 05416 | Viral myocarditis | 2 |
| 04370 | VEGF signalling pathway | 2 |
| 04151 | PI3K-Akt signalling pathway | 3 |
| 05120 | Epithelial cell signalling in Helicobacter pylori infection | 2 |
| 05100 | Bacterial invasion of epithelial cells | 2 |
| 04012 | ErbB signalling pathway | 2 |
| 04540 | Gap junction | 2 |
| 04912 | GnRH signalling pathway | 2 |
| 04066 | HIF-1 signalling pathway | 2 |
| 04020 | Calcium signalling pathway | 2 |

**Table 3**: KEGG Pathways (GO) in STRING analysis

**Table 4**: List of 54 genes yielded using K-means clustering method in STRING to identify groups within 181 genes interacting with TGF- β and EGFR.

| \| FLOT2 \| \| --- \| \| TRAF2 \| \| FYN \| \| TRADD \| \| BSG \| \| PLIN1 \| \| FLOT1 \| \| TNFRSF1A \| \| CAV2 \| \| TNF \| \| NOSTRIN \| \| CAV1 \| | \| FN1 \| \| --- \| \| LAMC1 \| \| ALB \| \| C4A \| \| SRC \| \| BMP4 \| \| ITGB1 \| \| POSTN \| \| TGFBR1 \| \| **TGFB1** \| \| ACVRL1 \| \| SMAD9 \|   PDGFRA | \| ENG \| \| --- \| \| SMAD2 \| \| SMAD3 \| \| GRB2 \| \| PTRF \| \| STAT3 \| \| HSP90AA1 \| \| **EGFR** \| \| EGF \| \| SOS1 \| \| SHC1 \| \| AKT1 \| \| INS \| | \| PTPN1 \| \| --- \| \| IRS1 \| \| CNN2 \| \| LCP1 \| \| FSCN1 \| \| WNT3A \| \| LRP6 \| \| AXIN1 \| \| ACAN \| \| TNC \| \| NCAN \| \| DNM2 \| \| NOS3 \| |
| --- | --- | --- | --- | --- | --- | --- | --- | --- | --- | --- | --- | --- | --- | --- | --- | --- | --- | --- | --- | --- | --- | --- | --- | --- | --- | --- | --- | --- | --- | --- | --- | --- | --- | --- | --- | --- | --- | --- | --- | --- | --- | --- | --- | --- | --- | --- | --- | --- | --- | --- | --- | --- | --- |

**Table 5:** Mean expression and standard deviation of Immunohistochemistry data as per different Grades and Stages

| GENES |  | | Normal | Stage1 | Stage 2 | Stage 3 | Stage 4 |
| --- | --- | --- | --- | --- | --- | --- | --- |
| POSTN | | Mean | 0.4 | 1.222 | 1.6 | 1.778 | 1.571 |
|  |  | Std. Dev. | 0.5164 | 0.9718 | 1.183 | 1.202 | 1.272 |
| CAV1 | | Mean | 0.4 | 1.667 | 1.667 | 2.111 | 2.286 |
|  |  | Std. Dev. | 0.5164 | 0.866 | 1.345 | 1.364 | 1.38 |
| TENASCIN | | Mean | 0.2 | 1.111 | 1.533 | 2.111 | 1.143 |
|  |  | Std. Dev. | 0.4216 | 0.7817 | 0.9904 | 0.7817 | 1.069 |
| FSCN1 | | Mean | 0.3 | ---- | 2.2 | 2.444 | 2.192 |
|  |  | Std. Dev. | 0.483 | ---- | 1.095 | 0.527 | 0.801 |

| GENES |  | | Normal | Grade 1 | Grade 2 | Grade 3 |
| --- | --- | --- | --- | --- | --- | --- |
| POSTN | | Mean | 0.4 | 0.8889 | 1.5 | 2.8 |
|  |  | Std. Dev. | 0.5164 | 0.4714 | 1.168 | 0.9189 |
| CAV1 | | Mean | 0.4 | 2.111 | 1.167 | 2.3 |
|  |  | Std. Dev. | 0.5164 | 1.231 | 1.337 | 0.8233 |
| TENASCIN | | Mean | 0.2 | 1.167 | 1.333 | 2.3 |
|  |  | Std. Dev. | 0.4216 | 0.7071 | 0.9847 | 0.9487 |
| FSCN1 | | Mean | 0.3 | 2 | 2.55 | 1.5 |
|  |  | Std. Dev. | 0.483 | 0.9075 | 0.5104 | 0.7071 |
